# Association of Parity With ASCVD Risk Factors Among South Asian Women: The MASALA Study

**DOI:** 10.1016/j.jacadv.2026.102647

**Published:** 2026-03-17

**Authors:** Adhya Mehta, Olayinka Agboola, Alka M. Kanaya, Namratha Kandula, Michael Blaha, Mahmoud Al Rifai, Garima Sharma, Jaideep Patel

**Affiliations:** a Department of Cardiology, Inova Health System, Falls Church, Virginia, USA; b Department of Medicine, University of California, San Francisco, California, USA; c Department of Preventive Medicine, Northwestern University, Chicago, Illinois, USA; d South Asian Cardiovascular Health Initiative, Johns Hopkins Ciccarone Center for the Prevention of Heart Disease, Baltimore, Maryland, USA; e Department of Cardiology, Houston Methodist DeBakey Heart & Vascular Center, Houston, Texas, USA

**Keywords:** coronary artery calcium, MASALA study, South Asian women population

**What is the clinical question being asked?**

What is the association of multiparity with incident coronary artery calcium and cardiovascular risk factors in South Asian women?

**What is the main finding?**

In South Asian women, multiparity was associated with higher blood pressure and low-density lipoprotein cholesterol over a 5-year follow-up period.

South Asian adults have a higher risk of atherosclerotic cardiovascular disease (ASCVD) events compared to other racial/ethnic groups, attributed to a higher prevalence of traditional risk factors (RFs). Specific to women, adverse pregnancy outcomes are associated with future ASCVD risk. Limited data exist on the interplay of multiparity and the development of subclinical atherosclerosis in South Asian (SA) women.^1^ Prevention guidelines support the use of coronary artery calcium (CAC) scoring to refine ASCVD risk. Parity has been linked to a higher prevalence of significant CAC, whereas the use of CAC in conjunction with standard risk estimators can restratify preventive efforts in SA adults.^1^

Given the associations of multiparity and ASCVD risk factors, we sought to evaluate the relationship between parity, cardiovascular risk factors, and CAC in SA women.

## Methods

The MASALA (Mediators of Atherosclerosis in South Asians Living in America) study protocol have been described previously.^2^ This study complies with human studies committees and is approved by UCSF IRB#10-00353 and Northwestern IRB#iSTU00019837. This is a community-based, prospective cohort of 906 SAs (48% women), aged 40 to 84 years without known ASCVD events. Baseline assessments were completed between 2010 to 2013, with follow-up between 2015 to 2018. We included women who completed exam 1 with complete RF measurements and CAC score (n = 415) and the subgroup of women with follow-up at exam 2 (n = 335).

Parity was defined as the number of live births and categorized as 0 to 1 and ≥2. Risk factors included exercise capacity, body mass index, waist circumference, systolic blood pressure (BP) (SBP), diastolic BP (DBP), fasting plasma glucose (FBS), total cholesterol, low-density lipoprotein cholesterol (LDL-C), high-density lipoprotein cholesterol, and triglycerides. Change in RFs was defined as the absolute difference between the RF at follow-up and baseline (normally distributed). CAC was assessed using noncontrast cardiac computed tomography scans, calculated at baseline and follow-up. As the distribution of this variable was skewed, it was log-transformed as log (CAC change+1). We defined incident CAC as the presence of any CAC (CAC>0) at exam 2 in a participant with no baseline CAC (CAC = 0) at exam 1.

Baseline demographic characteristics and ASCVD RFs were presented as percentages for categorical variables, mean (SDs) for uniformly distributed continuous variables, and median (IQRs) for nonuniformly distributed continuous variables.

Multivariable Tobit linear regression models were used to assess the association between parity and RFs and CAC score (baseline and change), log transformed as ln (CAC+1). Models were adjusted for age, family income, education, antihypertensive medication use, glucose-lowering medication use, and statin use. In a sensitivity analysis, models were further adjusted for body mass index, waist circumference, cigarette smoking, DASH (Dietary Approaches to Stop Hypertension) diet adherence score, physical activity, estimated glomerular filtration rate, LDL-C, high-density lipoprotein cholesterol, total cholesterol, triglycerides, SBP, DBP, and FBS. In another sensitivity analysis, parity was modeled as a continuous variable. A 2-sided *P* value was used with a statistical significance set as a *P* value of <0.05. The data were analyzed using STATA (version 16) (StataCorp LLC).

## Results

Among the 415 SA women (mean 54y, SD±9y), 38 (9.2%) had ≤1 live birth, and 377 had ≥2 live births. Both parity groups were comparable across sociodemographic, anthropometric, and baseline clinical variables except for FBS, which was higher among women with 1 or no prior live births (*P* = 0.04) (Table 1).

**Table 1:** Baseline Characteristics of the MASALA Female Population According to Parity, 2010-2013

	Parity		*P* Value
	0-1 (n = 38)	≥2 (n = 377)	
Age, y	52 (9)	54 (9)	0.14
Greater than bachelor’s degree, %	24 (63%)	195 (52%)	0.56
Annular income >$100,000, %	26 (70%)	226 (62%)	0.71
Any insurance, %	35 (92%)	343 (91%)	0.82
Birth region			0.23
India	28 (74%)	316 (84%)	
Other South Asian country	3 (8%)	24 (6%)	
Other diaspora country	7 (18%)	37 (10%)	
Total cholesterol, mg/dL	197 (43)	193 (35)	0.50
Low-density lipoprotein cholesterol, mg/dL	116 (34)	114 (31)	0.65
High-density lipoprotein cholesterol, mg/dL	56 (16)	56 (14)	0.99
Triglycerides, mg/dL	124 (57)	118 (50)	0.47
Statin medication use, %	8 (21%)	81 (21%)	0.95
Systolic blood pressure, mm Hg	121 (16)	123 (17)	0.44
Diastolic blood pressure, mm Hg	69 (9)	70 (10)	0.63
Hypertension, %	15 (39%)	131 (35%)	0.56
Antihypertensive medication use, %	11 (29%)	93 (25%)	0.56
Current smoking, %	0 (0%)	5 (1%)	0.76
Exercise, MET-min/week	472 (0-1710)	840 (315-1837)	0.59
Fasting glucose, mg/dL	106 (38)	97 (23)	**0.04**
Hemoglobin A1c, %	6.1 (1.0)	6.0 (0.7)	0.20
Diabetes, %	12 (32%)	77 (20%)	0.29
Glucose lowering medication use, %	6 (16%)	46 (12%)	0.52
Body mass index, kg/m^2^	27.0 (4.5)	26.0 (4.2)	0.19
Waist circumference, cm	89 (12)	89 (10)	0.94
Waist-to-hip ratio	0.86 (0.11)	0.86 (0.10)	0.59
Family history of coronary artery disease, %	17 (45%)	187 (50%)	0.57
Coronary artery calcium, AU			0.12
0	33 (86.8%)	287 (76.1%)	
1 – 99	5 (13.2%)	54 (14.3%)	
≥100	0 (0)	36 (9.6%)	

AU = Agatston unit; MET = metabolic equivalent of task.

**Bold** – significant value.

In adjusted models, no significant association between parity and baseline cardiovascular RFs or CAC (Table 2). When parity was modeled as a continuous variable, there was a significant inverse association with SBP (beta coefficient −1.34, 95% CI −2.58 to −0.11) and triglycerides (beta coefficient −4.35; 95% CI: −8.54 to −0.16).

**Table 2:** Multivariate-Adjusted Beta Coefficient (95% CI) for the Association of Parity and Baseline Cardiovascular Risk Factors and Coronary Artery Calcium Score, MASALA Study

	0-1 Births (n = 38)	≥2 Births (n = 377)
Exercise, MET-min/week^a^	1.00 (ref)	1.19 (−0.04 to 2.42)
Body mass index, kg/m^2^^a^	1.00 (ref)	−0.07 (−2.41 to 0.47)
Waist circumference, cm^a^	1.00 (ref)	−0.14 (−4.07 to 3.78)
Systolic blood pressure, mm Hg^a^	1.00 (ref)	0.59 (−4.37 to 5.55)
Diastolic blood pressure, mm Hg^a^	1.00 (ref)	0.99 (−1.93 to 3.92)
Fasting plasma glucose, mg/dL^a^	1.00 (ref)	−8.70 (−21.39 to 4.00)
Total cholesterol, mg/dL^a^	1.00 (ref)	−6.48 (−21.05 to 8.09)
Low-density lipoprotein cholesterol, mg/dL^a^	1.00 (ref)	−7.73 (−25.89 to 10.43)
High-density lipoprotein cholesterol, mg/dL^a^	1.00 (ref)	−4.36 (−15.42 to 6.70)
Triglycerides, mg/dL^a^	1.00 (ref)	−0.14 (−5.37 to 5.08)
Coronary artery calcium score, AU^a^	1.00 (ref)	2.33 (−0.17 to 4.84)
Coronary artery calcium score, AU^b^	1.00 (ref)	2.12 (−0.79 to 5.04)

Baseline characteristics were compared using the analysis of variance for continuous variables and the chi-square test for categorical variables.

Coronary artery calcium score was log-transformed as ln(CAC+1).

a Models are adjusted for age, income, education, antihypertensive medication use, glucose-lowering medication use, and statin medication use.

b Model adjusted for body mass index, waist circumference, cigarette smoking, DASH diet adherence score, physical activity, estimated glomerular filtration rate, low-density lipoprotein cholesterol, high-density lipoprotein cholesterol, total cholesterol, triglycerides, systolic blood pressure, diastolic blood pressure, and plasma glucose.

Of the 335 women who returned for exam 2, 219 women maintained CAC = 0 over the follow-up period (4.8 ± 0.8 years), whereas 39 women had incident CAC>0. There was a significant positive association between parity and change in SBP (beta coefficient 8.89; 95% CI: 3.09-14.69), change in DBP (beta coefficient 4.10; 95% CI: 0.90-7.30) and LDL-C (beta coefficient 8.92, 95% CI 0.54-17.31).

## Discussion

In this study, multiparity was associated with lower FBS at baseline and changes in BP and LDL-C. Although multiparity was associated with significantly higher CAC at baseline, no significant association was found between parity and incident CAC or change in CAC over 5 years of follow-up.

We observed a significant association of parity with LDL-C levels. Pregnancy induces metabolic changes and oxidative stress that affect lipid metabolism; multiparity may lead to cumulative effects with long-term consequences for lipid profiles. These associations may be amplified in SA women due to a predisposition to central adiposity and poor dietary patterns. Ethnic differences in lipid concentration during pregnancy and the postpartum period have been reported.^3^

Our study observed a positive association between parity and SBP and DBP. Similarly, a study among Sudanese women found a significant association of parity with high BP.^4^ Hypertensive disorders of pregnancy affect ∼54/1,000 live births in SA women.^1^ In a prior MASALA analysis, the relative prevalence of CAC>100 was the highest for a DBP of approximately 75 to 95 mm Hg among those not on antihypertensive therapy (*P* < 0.01)^1^, suggesting closer monitoring and management of DBP among SA multiparous women.

We did not find a significant association between parity and baseline or change in CAC, respectively, which may be due to short-term follow-up in a small sample of younger participants. Indeed, the prevalence of any detectable CAC in SA women is highest beyond age 65 years.^5^ Notably, the absence of traditional RFs is associated with maintenance of CAC = 0^5^ and likely contributes to the low prevalence of incident CAC in follow-up. In other studies that did not include SA women and had longer follow-up (10 years), a greater progression and incidence of CAC have been described among women with ≥4 births, lending credence to the detection of CAC to guide preventive medications to mitigate future ASCVD morbidity and mortality.

Notable limitations include a small sample size composition of the MASALA cohort (primarily Asian Indian immigrants)—limiting generalizability, considering better RF control compared to other SA populations. Despite sharing similar genetic and cultural backgrounds, SA differ in health-seeking behaviors and environmental factors.^1^ Lastly, residual confounding is possible.

## Conclusions

SA women with multiparity of 2 or more live births had higher baseline CAC and were found to have a greater change in BP and LDL after 5 years of follow-up. These results highlight variability in the association between parity and cardiovascular risk across different populations and the importance of monitoring ASCVD RFs in SA women, given their increased predisposition to early ASCVD and adverse pregnancy outcomes.

## Funding support and author disclosures

The authors have reported that they have no relationships relevant to the contents of this paper to disclose.
